# 

**Published:** 1905-04

**Authors:** 


April, 1905. Vol. XXVI. No. 4.

### THE INTERNATIONAL DENTAL JOURNAL.

LIBRARY
SURGEON GENERAL'S OFFICE
APR. 7-1905

LIBRARY
INDEXED
S. G. O.

JAMES TRUMAN, D.D.S., Editor,

PHILADELPHIA.

Collaborator: LAWRENCE W. BAKER, D.M.D.,
BOSTON.

### Summary of Contents.

(*For details, see p. 2.*)

ORIGINAL COMMUNICATIONS.

REVIEWS OF DENTAL LITERATURE.

REPORTS OF SOCIETY MEETINGS.

EDITORIAL.

DOMESTIC CORRESPONDENCE.

FOREIGN CORRESPONDENCE.

CURRENT NEWS.

*$2.50 a Year, in Advance. Single Copies, 25 Cents.*

PUBLISHED MONTHLY BY

The International Dental Publication Company,

227–231 SOUTH SIXTH ST., PHILADELPHIA.

EDITORIAL OFFICE, 4505 CHESTER AVENUE, PHILADELPHIA, PA.

Printed by J. B. Lippincott Company, Philadelphia.

Entered at the Post-Office at Philadelphia, and admitted for transmission through the mails at second-class rates.

